# The effects of pulmonary rehabilitation in patients with non-cystic fibrosis bronchiectasis: protocol for a randomised controlled trial

**DOI:** 10.1186/1471-2466-10-5

**Published:** 2010-02-02

**Authors:** Annemarie L Lee, Nola Cecins, Catherine J Hill, Anne E Holland, Linda Rautela, Robert G Stirling, Phillip J Thompson, Christine F McDonald, Sue Jenkins

**Affiliations:** 1 Physiotherapy, Melbourne School of Health Sciences, The University of Melbourne, Melbourne, Australia; 2 Department of Physiotherapy, Alfred Health, Melbourne, Australia; 3 Institute for Breathing and Sleep, Melbourne, Australia; 4 Physiotherapy Department, Sir Charles Gairdner Hospital, Perth, Australia; 5 School of Physiotherapy and Curtin Health Innovation Research Institute, Curtin University of Technology, Perth, Australia; 6 Lung Institute of Western Australia and Centre for Asthma, Allergy and Respiratory Research, University of Western Australia, Perth, Australia; 7 Department of Physiotherapy, Austin Health, Melbourne, Australia; 8 School of Physiotherapy, La Trobe University, Melbourne, Australia; 9 Department of Allergy, Immunology and Respiratory Medicine, Alfred Health, Melbourne, Australia; 10 Department of Medicine, Monash University, Melbourne, Australia; 11 Department of Respiratory Medicine and Sleep, Austin Health, Melbourne, Australia

## Abstract

**Background:**

Non-cystic fibrosis bronchiectasis is characterised by sputum production, exercise limitation and recurrent infections. Although pulmonary rehabilitation is advocated for this patient group, its effects are unclear. The aims of this study are to determine the short and long term effects of pulmonary rehabilitation on exercise capacity, cough, quality of life and the incidence of acute pulmonary exacerbations.

**Methods/Design:**

This randomised controlled trial aims to recruit 64 patients with bronchiectasis from three tertiary institutions. Participants will be randomly allocated to the intervention group (supervised, twice weekly exercise training with regular review of airway clearance therapy) or a control group (twice weekly telephone support). Measurements will be taken at baseline, immediately following the intervention and at six and 12 months following the intervention period by a blinded assessor. Exercise capacity will be measured using the incremental shuttle walk test and the six-minute walk test. Quality of life and health status will be measured using the Chronic Respiratory Questionnaire, Leicester Cough Questionnaire, Assessment of Quality of Life Questionnaire and the Hospital Anxiety and Depression Scale. The rate of hospitalisation will be captured as well as the incidence of acute pulmonary exacerbations using a daily symptom diary.

**Discussion:**

Results from this study will help to determine the efficacy of supervised twice-weekly pulmonary rehabilitation upon exercise capacity and quality of life in patients with bronchiectasis and will contribute to clinical practice guidelines for physiotherapists in the management of this population.

**Trial registration:**

This study protocol is registered with ClinicalTrials.gov (NCT00885521).

## Background

Bronchiectasis not related to cystic fibrosis (non-CF bronchiectasis) is characterised by permanent dilatation of the airways arising from bronchial inflammation and infection [1]. Predominant symptoms include cough with sputum production, dyspnoea and fatigue [2-4]. This clinical profile contributes to progressive deconditioning, which gives rise to reduced exercise tolerance and diminished health-related quality of life (HRQoL) [2,5,6]. Patients with bronchiectasis suffer from recurrent acute exacerbations, which may require hospitalisation [7,8]. This clinical course of bronchiectasis imposes considerable economic cost to the healthcare system [9,10]. More importantly, the incidence of pulmonary exacerbations is a prognostic predictor in bronchiectasis, with progressive airway damage and decline in respiratory function greater in those experiencing a higher incidence of acute exacerbations [11].

Medical intervention has limited impact on disease progression or exercise capacity and evidence regarding the cost effectiveness of any therapeutic approach in bronchiectasis is scant [1]. Exercise and self management training, as a component of a pulmonary rehabilitation (PR) program is a well-established and effective intervention for patients with chronic obstructive pulmonary disease (COPD), leading to reductions in the incidence of acute exacerbations and reduced health care utilisation as well as improvements in exercise tolerance and HRQoL [12-16]. Although international guidelines for PR recommend the inclusion of patients with bronchiectasis within existing programs [12], there is a lack of evidence supporting this practice [17]. One study has compared exercise training with or without inspiratory muscle training to standard medical care [18]. While significant short-term improvement in exercise capacity was demonstrated, benefits in HRQoL were only achieved with a combination of whole body exercise and inspiratory muscle training. The clinical significance of these findings is unclear, as the administered intervention differed from current recommendations for PR [12,19]. Although inspiratory muscle training was applied as a form of airway clearance therapy (ACT) in this study [18], no beneficial effects on ease of sputum expectoration were demonstrated. The long term benefits of PR which incorporates exercise training and self management in ACT compared to standard medical care, on exercise capacity and dimensions of HRQoL, including fatigue, anxiety and depression remains unknown. Furthermore, evaluation of the efficacy of such an intervention on the incidence of acute pulmonary exacerbations of bronchiectasis may provide evidence to determine best practice guidelines in this patient population.

The primary aims of this study are to (1) determine the short and long term effects of an eight-week PR program consisting of supervised exercise training and self management education in ACT compared to standard care on exercise capacity and HRQoL; and (2) establish the effect of this intervention on the incidence of acute exacerbations over a 12 month period in patients with bronchiectasis. We hypothesise that patients who complete PR will have a higher exercise capacity and HRQoL compared to standard care and that these benefits will be sustained for up to six months. PR will also reduce the incidence of pulmonary exacerbations over 12 months.

## Methods/Design

### Study design

This multi-centre, randomised controlled trial (RCT) will be conducted at Alfred Health and Austin Health, Melbourne, the Lung Institute of Western Australia and Sir Charles Gairdner Hospital, Perth, Australia.

### Participants

#### Inclusion/exclusion criteria

To be eligible for enrolment, participants must be aged ≥ 18 years and have a diagnosis of bronchiectasis that is not attributable to CF, confirmed radiologically on high resolution computed tomography. They must be clinically stable, with no evidence of an exacerbation of bronchiectasis or changes in medical therapy in the previous four weeks [7]. Other inclusion criteria comprise of exertional dyspnoea (Modified Medical Research Council (MMRC) score ≥ 1) [20] and a history of at least two exacerbations per year over the past two years.

Participants will be excluded if they have 1) smoking history ≥ 10 pack years or physician diagnosis of COPD [21]; 2) a clinical diagnosis of asthma [22]; 3) interstitial lung disease (clinical/radiological diagnosis); 4) medical conditions which could place the individual at risk during exercise testing or training (eg. unstable cardiovascular disease) or conditions that may restrict the participant's ability to exercise (eg. severe orthopaedic or neurologic impairments; 5) participation in a PR program within the last 12 months.

#### Sample size

To detect a true difference in the primary outcome measures of maximal exercise capacity and HRQoL, a total of 36 subjects (18 per group) will be required. This is based on the 80% probability of detecting statistically and clinically significant differences in maximal exercise capacity (incremental shuttle walk test [ISWT]) [18] and HRQoL (Chronic Respiratory Questionnaire [CRQ] total score), according to pilot data from 33 patients with bronchiectasis who completed PR at Alfred Health. To detect a true difference for the secondary outcome measure of the incidence of acute exacerbations, a total of 64 subjects will be required. This is based on the 80% probability of detecting a true difference in the number of exacerbations between groups is 1.0 exacerbation, with a SD of 1.4 exacerbations [11].

#### Recruitment and randomisation

**Figure 1:**
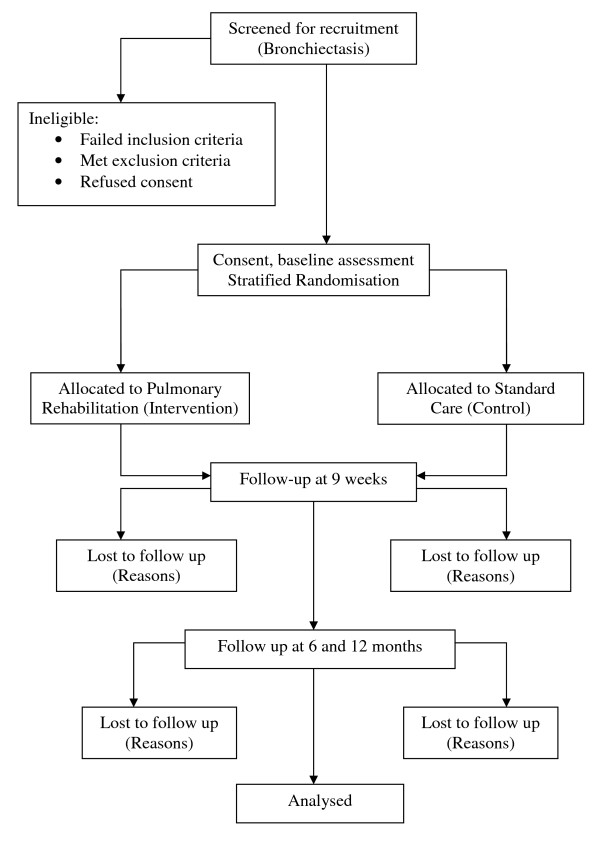
**Flow of patients through the study**.

The flow of participants through the study will reflect the recommendations from the Consolidated Standards of Reporting Trials statement [23] and is outlined in Figure 1. Participants will receive written and verbal information explaining the study and written consent will be obtained from all participants. The Human Research Ethics Committee of Alfred Health, Austin Health and Sir Charles Gairdner Hospital approved the study and the protocol is registered with ClinicalTrials.gov (NCT00885521).

Participants will be screened from the Lung Institute of Western Australia (LIWA) database of individuals with bronchiectasis or during outpatient clinic visits to Physiotherapy or Respiratory Medicine at Alfred Health, Austin Health and Sir Charles Gairdner Hospital, by the study investigators. If eligible, the study investigators will approach the participant to explain the study and gain written, informed consent. It will be explained that they may withdraw from the study at any time. Within strata, participants will be randomly assigned to receive either standard care alone or PR. The strata are sputum quantity (small volume: up to one tablespoon per day, large volume: > one tablespoon per day) and the site of randomisation. The randomisation will be completed by the creation of a random numbers table and individual group assignment will be kept in sealed opaque envelopes. Following baseline assessments, a physiotherapist will collect the appropriate envelope after confirming sputum quantity. A computer-generated randomised order for performing the field walking tests for each participant will be determined before baseline measures and adhered to throughout the study.

### Intervention

#### Standard care group

These participants will receive instruction and review of ACT provided by a senior physiotherapist. This process is consistent with current clinical management at each recruitment site. Each participant will be provided with written information about bronchiectasis [24] and education regarding self-management of the condition. Participants who have not been previously instructed in any ACT will be taught the active cycle of breathing technique [25]. Participants previously instructed in other forms of ACT (eg. positive expiratory pressure therapy, oscillating positive expiratory pressure therapy) will have their technique reviewed, corrected if necessary and encouraged to continue with this corrected technique for the study duration. Instructions in ACT will be given during the baseline assessment. These participants will not receive any supervised exercise training but will be informed at the baseline assessment that accumulating 30 minutes of moderate intensity physical activity on most days of the week is the standard recommendation for health benefits. During the eight-week intervention period, participants will be contacted by telephone twice weekly. The purpose of these calls is to answer any questions the participant may have regarding performance of their ACT and to provide support and general health advice using a standardised script. This is an accepted strategy to control for clinician contact in trials involving disabled patients with chronic lung disease and has been previously used in patients with interstitial lung disease [26]. No specific discussion related to physical activity will be undertaken during these telephone calls.

#### Pulmonary rehabilitation group

Participants randomised to the PR group will receive exercise training and regular instruction in self-management of ACT. No additional formal education sessions will be included. Participants will receive the identical instruction in ACT as described for the standard care group. Self management strategies to encourage regular adherence with ACT will also be implemented. Once a week at the conclusion of a PR session, participants will record their daily sputum volume on a 100 mm visual analogue scale and have their ACT reviewed by a senior physiotherapist. This will include observation of the participant's technique with correction where necessary and discussion of any concerns or problems the participant is experiencing with the self-management program.

The eight-week exercise training program will comprise of two supervised exercise classes at the institutions each week with a home walking program on two additional days per week. The exercise program will follow recent guidelines for exercise prescription in chronic lung disease [19]. This will comprise of 30 minutes of endurance exercise at each supervised session, including walking and cycling. Initial walking intensity will be prescribed at 75% of the maximal work rate achieved on the ISWT while cycling intensity will be based on data obtained from the 6-minute walk test (6MWT) [27]. For participants unable to tolerate continuous exercise, interval training will be used. Participants will also complete a circuit of upper limb endurance exercises and functional strength training for the lower limbs. An experienced physiotherapist will monitor participants during the exercise classes and progress the exercise within symptom tolerance of dyspnoea and fatigue based on the 0 to 10 Borg scale [28]. Supplemental oxygen will be provided during training if necessary to achieve an oxygen saturation >85% [19]. Following the establishment of a safe, supervised exercise regimen, a home exercise program will be prescribed, with participants encouraged to walk for 30 minutes at an intensity similar to that achieved in the supervised sessions. Participants will record these walking sessions in a home exercise diary. Attendance at 12 out of 16 supervised sessions will be considered completion of the exercise intervention. At the completion of the intervention period, participants will be instructed to continue with their home exercise program three to five times per week and to record these sessions in their exercise diary.

### Outcome measures

All outcome measures will be recorded at baseline, immediately following the intervention period (nine weeks) and at six and 12 months post completion of the intervention (Figure 1). Baseline data collection will include age, gender, body mass index, usual ACT if previously prescribed and MMRC scale for dyspnoea [20]. The number of acute pulmonary exacerbations per year in the previous two years will also be collated. An exacerbation will be defined as an increase in clinical signs and symptoms requiring alteration of medication, including antibiotic therapy according to participant recall and medical record review.

### Primary outcome measures

The primary outcome measures are maximal exercise capacity and HRQoL. An independent assessor, blinded to group allocation will perform all outcome assessments following the intervention period (nine weeks, six and 12 months) which will be conducted over a two day period at each time point.

**a) Maximum exercise capacity **will be measured with the ISWT [29]. To allow for a learning effect, this test will be performed twice, with a 30 minute rest period in between each test. The primary outcome is the greatest distance walked. The ISWT has demonstrated a moderate to strong correlation with peak rate of oxygen uptake [30], has shown responsiveness to change following PR [12] and has been used in patients with non-CF bronchiectasis [18].

b) **HRQoL **will be measured using the CRQ (self-administered version) [31-33]. This disease-specific questionnaire has demonstrated responsiveness to change following PR [33]. The CRQ consists of 20 questions and assesses the domains of dyspnoea during activities of daily living, fatigue, emotional function and mastery, using a 7-point Likert scale and provides a total HRQoL score. A higher score indicates better HRQoL.

### Secondary outcome measures

a) **Cough specific HRQoL **will be measured using the Leicester Cough Questionnaire (LCQ). The LCQ measures the physical, psychological and social impact of chronic cough [34]. It consists of 19 items with responses based on a 7-point Likert scale and has been validated in patients with non-CF bronchiectasis [35]. A higher score indicates less impact on HRQoL.

b) **Functional exercise capacity **will be measured using the 6MWT [36]. The distance walked (6 minute walk distance, [6MWD]) has demonstrated strong correlation with peak rate of oxygen uptake and maximum work rate in patients with chronic respiratory disease [27,30]. To allow for familiarisation (or learning effect), two tests will be conducted using a standardised protocol [36] with the greatest distance recorded. The 6MWT is responsive to PR [12].

3) **Anxiety and depression **will be measured with the Hospital Anxiety and Depression Scale (HADS). This questionnaire is designed to detect and measure the severity of anxiety and depression [37]. It consists of a series of 14 statements, with responses based on a 4-point Likert scale. The HADS is self-administered and has demonstrated responsiveness to PR [38]. A higher score is indicative of greater anxiety or depression.

4) **HRQoL utility **will be measured using the Assessment of Quality of Life (AQOL) Questionnaire [39]. This measure reflects a patient's HRQoL across five dimensions: illness, independent living, social relationships, physical senses and psychological well being [39]. It is comprised of 15 questions, with the life-death utility scale score ranging from 1.00 (reflecting best possible HRQoL equivalent state) and 0.00 (worse than death equivalent state), which is then used to calculate quality adjusted life years [39]. The AQOL has been validated in different populations, but has not been previously used in individuals with bronchiectasis.

5) Symptom diary. All participants will complete a daily diary for the duration of the study, with the diaries collected at the conclusion of each assessment. Participants will record a score for quantity and colour of secretions expectorated each day. In addition, participants will also record if they have at least four of the following clinical signs and symptoms suggestive of an acute exacerbation of bronchiectasis [7] over two consecutive days:

• Increase in sputum quantity

• Change in sputum colour

• Change in sputum viscosity

• Increase in cough

• Increase in dyspnoea

• Increase in fatigue/lethargy

• Fever (self-reported)

• Episode of haemoptysis

Participants will also record occasions of unplanned review by their general practitioner or hospital admissions (duration of hospitalisation) together with the prescription of antibiotic therapy (days required) secondary to a pulmonary exacerbation of bronchiectasis in their symptom diary. These events will be cross-checked with hospital and general practitioner medical records at the conclusion of the study. Adjudication of a respiratory exacerbation will be completed by an independent investigator.

Spirometry will be performed in accordance with the American Thoracic Society guidelines [40]. The highest value for forced expiratory volume in one second (FEV_1_) and forced vital capacity (FVC) obtained from three reproducible trials will be recorded and compared to predicted normal values. This will provide a measure of the severity of airflow limitation and will be used to assess stability of lung function over the duration of the follow-up period of the study.

### Statistical analysis

Data will be entered into SPSS (version 17.0; SPSS Inc; Chi) for analysis. Descriptive statistics will be used to examine the distribution and data that are not normally distributed will be transformed or analysed using non-parametric tests. Analyses will be conducted using an intention to treat principle. Repeated measures analysis of variance with planned comparison will be used to compare all continuous variables that are normally distributed. Significant results will be analysed using post hoc tests. Categorical variables will be analysed using the chi square statistic. Relationships between outcome variables will be analysed using correlation coefficients. The alpha level for all statistical analyses will be set at 0.05.

## Discussion

Bronchiectasis has a heterogeneous clinical profile, secondary to the multiple aetiologies from which it may originate [1,7]. Although the precise global prevalence is unknown, bronchiectasis remains a cause of excessive morbidity [8]. In the current climate of limited health resources, it is important to provide interventions which not only contribute to improved HRQoL, but positively impact on disease progression and prognosis.

This research will be the first intervention study evaluating the short and long term efficacy of PR, including exercise training and self-management in ACT compared to standard care, in patients with non-CF bronchiectasis. Currently, progression of respiratory symptoms is evident in this heterogenous population despite optimal medical intervention [8]. We recently found that symptoms of chronic cough, sputum production and fatigue, based on the St George's Respiratory Questionnaire were associated with reduced exercise capacity in patients with mild to moderate bronchiectasis [41]. This implies that strategies such as a PR program which incorporates self management and adherence to ACT may have an equally positive impact on both diminished exercise capacity and clinically relevant dimensions of HRQoL, both of which will be important outcomes for all patients with bronchiectasis. Given the relationship between exacerbation rate and decline in pulmonary function [11], it would be highly significant if PR were to impact on the incidence of acute exacerbations in bronchiectasis. As both declining respiratory function and reduced physical activity are associated with increased mortality in this population [42], achieving a reduction in the incidence of pulmonary exacerbations would result in PR being one of the few available treatments with the potential to modify the disease course and prognosis in bronchiectasis and may be an inexpensive complement to existing medical care.

The results of this study will assist in the development of physiotherapy guidelines for the treatment of patients with bronchiectasis in clinical practice. While a recent survey revealed that the majority of physiotherapists in Australia and New Zealand routinely prescribed a form of ACT for patients with bronchiectasis, recommendations for exercise training were inconsistent [43]. In addition, no specific guidelines exist to guide clinicians in exercise prescription and the role of self management in ACT for patients with bronchiectasis. If the study findings are positive, the application of PR should be routinely instituted for all patients.

## Abbreviations

ACT: Airway clearance therapy; AQOL: Assessment of Quality of Life; CF: Cystic fibrosis; COPD: Chronic obstructive pulmonary disease; CRQ: Chronic Respiratory Questionnaire; FEV_1_: Forced expiratory volume in one second; FVC: Forced vital capacity; HADS: Hospital Anxiety and Depression Scale; HRQoL: Health related quality of life; ISWT: Incremental shuttle walk test; LCQ: Leicester Cough Questionnaire; MMRC: Modified Medical Research Council; PR: Pulmonary rehabilitation; RCT: Randomised controlled trial; 6MWD: Six-minute walk distance; 6MWT: Six-minute walk test.

## Competing interests

The authors declare that they have no competing interests.

## Authors' contributions

AL, NC, AH, CH, CM and SJ designed the trial protocol. AL, NC, AH, CH, PT, RS, CM and SJ procured the study funding. AL drafted the manuscript and NC, AH, CH, LR, RS, PT, CM and SJ contributed to the manuscript. All authors read and approved the final manuscript.

## Pre-publication history

The pre-publication history for this paper can be accessed here:

http://www.biomedcentral.com/1471-2466/10/5/prepub
